# The *k*-Robinson–Foulds Dissimilarity Measures for Comparison of Labeled Trees

**DOI:** 10.1089/cmb.2023.0312

**Published:** 2024-04-22

**Authors:** Elahe Khayatian, Gabriel Valiente, Louxin Zhang

**Affiliations:** ^1^ Department of Mathematics, National University of Singapore, Singapore, Singapore.; ^2^ Department of Computer Science, Technical University of Catalonia, Barcelona, Spain.

**Keywords:** *k*-Robinson–Foulds dissimilarity, labeled trees, phylogenetic trees

## Abstract

Understanding the mutational history of tumor cells is a critical endeavor in unraveling the mechanisms that drive the onset and progression of cancer. Modeling tumor cell evolution with labeled trees motivates researchers to develop different measures to compare labeled trees. Although the Robinson–Foulds (RF) distance is widely used for comparing species trees, its applicability to labeled trees reveals certain limitations. This study introduces the *k*-RF dissimilarity measures, tailored to address the challenges of labeled tree comparison. The RF distance is succinctly expressed as *n*-RF in the space of labeled trees with *n* nodes. Like the RF distance, the *k*-RF is a pseudometric for multiset-labeled trees and becomes a metric in the space of 1-labeled trees. By setting *k* to a small value, the *k*-RF dissimilarity can capture analogous local regions in two labeled trees with different size or different labels.

## 1. INTRODUCTION

In the realm of evolutionary biology, trees serve as fundamental mathematical concepts, offering a versatile framework for modeling the evolution of various entities, including organisms, species, and genes. Beyond their application in understanding biological evolution, trees find practical utility in medical diagnosis within the health care domain. The diversity of tree models has given rise to the significant challenge of effectively comparing different trees to evaluate various inference methods. This challenge has spurred researchers to define robust measures within the space of targeted trees. For example, mutation/clonal trees are introduced to model tumor evolution. In this representation, nodes denote cellular populations and are labeled with the gene mutations present in those populations (Karpov et al., 2019; Schwartz and Schäffer, 2017).

The growth and metastasis of tumors varies from patient to patient. In addition, such variations are significant for cancer treatment. As a result, dissimilarity measures for mutation tree comparison have become a focus of recent research (DiNardo et al., 2020; Jahn et al., 2021; Karpov et al., 2019; Llabrés et al., 2021).

In earlier work on phylogenetic trees, various measures have been proposed to compare two phylogenetic trees. Some examples of such measures are Robinson–Foulds (RF) distance (Robinson and Foulds, 1981), nearest-neighbor interchange (NNI) (Li et al., 1996; Robinson, 1971), Quartet distance (Estabrook et al., 1985), and Path distance (Steel and Penny, 1993; Williams and Clifford, 1971). They are defined under the assumption that the involved trees share the same label set. Consequently, they may not be useful when applied to trees where all nodes are labeled, especially when using different label sets.

### 1.1. Related work on comparison of labeled trees

To overcome the constraints associated with the above-mentioned measures in the comparison of mutation trees, computational biologists have introduced new dissimilarity metrics for mutation trees. Some of these measures are Common Ancestor Set (CASet) distance (DiNardo et al., 2020), Distinctly Inherited Set Comparison (DISC) distance (DiNardo et al., 2020), and Multi-Labeled Tree Dissimilarity measure (Karpov et al., 2019). Although these distance measures enable efficient comparison of clonal trees, they are defined based on the assumption that mutations cannot occur more than once and mutations will not be lost in the course of tumor evolution. As a result, these metrics exhibit multiple limitations when applied to the comparison of trees used to model complex tumor evolution, wherein mutations may indeed occur multiple times and subsequently be lost.

Apart from the three measures discussed earlier, a few additional dissimilarity metrics have been introduced to facilitate the comparison of mutation trees, including Parent–Child distance (Govek et al., 2018) and Ancestor–Descendant distance (Govek et al., 2018). These measures are metric for “1-mutation” trees, in which nodes are each labeled by one distinct mutation.

There are also measures for mutation trees that are defined through generalization of popular measures that are used for phylogenetic trees. Here, researchers aim to extend the definition of an existing distance, which was mostly used to compare phylogenetic trees with mutation trees. For example, the generalized NNI (Jahn et al., 2021) is defined by some minor modifications of NNI. The other example is the Path distance (Govek et al., 2018). Although these measures are applicable to mutation trees, they are only well defined for mutation trees with the same label sets (Govek et al., 2018; Jahn et al., 2021).

The generalized RF (GRF) distance is another distance introduced recently (Llabrés et al., 2021; Llabrés et al., 2020). This measure is used not only to compare mutation trees or clonal trees but also enables the comparison of species trees and even phylogenetic networks. A useful property of GRF is that its value is significantly contributed by the intersection of clusters or clones of targeted trees. However, the intersection is not quantified in the RF distance, as one only checks whether two cluster or clones of the two involved trees are identical or not when the RF-distance between two trees is computed. As a result, the GRF has a better resolution than the RF distance (Llabrés et al., 2020).

There are some other generalizations of the RF distance, such as Bourque distance (Jahn et al., 2021). The measure is able to compare mutation trees with same or different label sets, and it has linear time complexity. However, like the above distances, it does not allow for multiple occurrences of mutations during the tumor history (Jahn et al., 2021). Other generalizations of the RF distance have also been proposed for gene trees (Briand et al., 2022; Briand et al., 2020).

The aforementioned dissimilarity measures do not apply to some evolutionary models, such as Dollo (Farris, 1977) and the Camin–Sokal model (Camin and Sokal, 1965). This is because mutations may get lost after they are gained in the Dollo model, and the same mutation may occur more than once during the tumor history in the Camin–Sokal model (Llabrés et al., 2020). As far as we know, the only measure introduced to address the problem is the Triplet-based Distance (Ciccolella et al., 2021). The distance allows to compare mutation trees in which nodes have nonempty subsets of mutations as their labels. In addition, it also allows multiple occurrences and losing of mutations during the tumor history (Ciccolella et al., 2021). Despite the applicability of the measure to the larger group of labeled trees, Triplet-based Distance does not apply to labeled trees in which multiple copies of a mutation is observed in the label of a single node.

### 1.2. Our contributions to tree comparison

In this study, we develop the *k*-RF dissimilarity measures designed for the comparison of labeled trees. They are first defined for 1-labeled trees (Section 3). Subsequently, we extend these measures to multiset-labeled trees (Section 5). We delve into the mathematical properties of the *k*-RF measures in Sections 4 and 5. In particular, *k*-RF is a metric for 1-labeled trees. We also assess the validity of the *k*-RF measures through comparisons with CASet, DISC, and GRF (Section 5), and the evaluation of their performance in the context of tree clustering (Section 6).

## 2. CONCEPTS AND NOTATIONS

A (directed) graph consists of a set of nodes and a set of (directed) edges. In graphs, each edge is a pair of distinct nodes. In directed graphs, each edge is a pair of ordered distinct nodes.

Let G be a (directed) graph. *V*(*G*) and *E*(*G*) are used to denote its node and edge set, respectively. If *G* is undirected, (*u,v*) will still be used to denote an edge between *u* and *v* with the understanding that (*u,v*) = (*v,u*). Let $u,v\in V\left(G\right)$. If $\left(u,v\right)\in E\left(G\right)$, we say that *u* and *v* are adjacent, the edge (*u,v*) is incident to *u* and *v*, or *u* and *v* are two endpoints of (*u,v*).

The degree of *v* is defined as the number of edges incident to *v*. In addition, if *G* is directed, the *indegree* and *outdegree* of *v* are defined as the number of edges (*x,y*) such that *y* = *v* and *x* = *v*, respectively. The nodes of degree 1 are called the *leaves* in an undirected graph, whereas the nodes of indegree 1 and outdegree 0 are called the *leaves* in a directed graph. We use *Leaf* (*G*) to denote the leaf set for *G*. Nonleaf nodes are called *internal nodes*.

A *path* of length *k* from *u* to *v* consists of a sequence of nodes $u_{0},u_{1},\ldots ,u_{k}$ such that $u_{0}=u$, $u_{k}=v$ and $\left(u_{i-1},u_{i}\right)\in E\left(G\right)$ for i=1,2,⋯,k. The *distance* from *u* to *v*, denoted as $d_{G}\left(u,v\right)$, is the length of the shortest paths from *u* to *v*, and it is set to ∞ if there is no path from *u* to *v*.

If *G* is undirected, $d_{G}\left(u,v\right)=d_{G}\left(v,u\right)$ for any $u,v\in V\left(G\right)$. The *diameter* of *G* is defined as $max_{u,v\in V\left(G\right)}d_{G}\left(u,v\right)$ and is denoted by diam(*G*). If *G* is directed, its diameter is defined as the diameter of its undirected version that has the node set *V*(*G*) and edge set $E\left(G\right)\cup \left\{\left(u,v\right)|\left(v,u\right)\in E\left(G\right)\right\}$.

### 2.1. Trees

A tree *T* is a graph in which there is a unique path between any two distinct nodes. A *binary* tree is a tree in which every internal node has degree 3. A *line tree* is a tree in which every internal node has degree 2. The number of leaves in a line tree is 2.

A directed tree is a directed graph that is a tree if we ignore the orientations of edges.

### 2.2. Rooted trees

A directed tree is called a rooted tree if it has a special root node from which the edges are directed away. In a rooted tree, indegree of each nonroot node is 1, which implies that there is exactly one path from its root to any other node.

Let *T* be a rooted tree, $u,v\in V\left(T\right)$ such that u≠v. We say *v* is a child of *u* and *u* is the parent of *v* if $\left(u,v\right)\in E\left(T\right)$. In general, we say *v* is a descendant of *u*, and *u* is an ancestor of *v* if *u* is in the unique path from *root*(*T*) to *v*. The set of all children, ancestors and descendants of *u* are denoted by $C_{T}\left(u\right)$, $A_{T}\left(u\right)$ and $D_{T}\left(u\right)$, respectively. Note that $u\notin A_{T}\left(u\right)$ and $u\notin D_{T}\left(u\right)$.

A rooted tree is called a binary rooted tree if the root has indegree 0 and outdegree 1, and every other internal node has indegree 1 and outdegree 2.

A rooted tree is called a rooted line tree if each internal node has exactly one child. A rooted tree is called a rooted caterpillar tree if the set of children of each internal node contains at most one internal node.

### 2.3. Labeled trees

Suppose *L* is a set and $P\left(L\right)$ denotes the set of all subsets of *L*. We say a tree or rooted tree *T* is labeled by subsets of *L* if *T* is equipped by a map $\ell :V\left(T\right)\to P\left(L\right)$, where $\cup_{v\in V\left(T\right)}\ell \left(v\right)=L$, and $\ell \left(v\right)\neq \emptyset$ for any $v\in V\left(T\right)$. In particular, we say *T* is 1-labeled on *L* if $\ell \left(v\right)$ is a singleton for each $v\in V\left(T\right)$, and ℓ is ingective. Moreover, for a 1-labeled tree *T* on *L* and $C\subseteq V\left(T\right)$, we define $L\left(C\right)=\left\{a\in L|\exists x\in C:\ell \left(x\right)=\left\{a\right\}\right\}$.

### 2.4. Phylogenetic and mutation trees

Let *X* be a finite taxon set. A phylogenetic tree (respectively, rooted phylogenetic tree) on *X* is a binary tree (respectively, binary rooted tree) in which only leaves are labeled by the elements of *X*, and two distinct leaves have different labels.

A mutation tree on a set *M* of mutations is a rooted tree in which nodes are labeled with nonempty subsets of *M*.

### 2.5. Dissimilarity measures for trees

Let T be a set of trees. A dissimilarity measure on T is a real function *d* such that $d\left(T\prime ,T\prime \prime \right)=d\left(T\prime \prime ,T\prime \right)\geq 0$ for any T′,T′′∈T. It captures the intuition that the more different two trees are, the higher their measure value is and vise versa. It is called a pseudometric if it satisfies triangle inequality condition. A pseudometric *d* is called a metric if $d\left(S,T\right)\neq 0$ unless *S* and *T* are the same trees.

## 3. THE *k*-RF MEASURE FOR 1-LABELED TREES

In this section, we first recall the definition of the RF distance and then present *k*-RF dissimilarity measures for 1-labeled trees for arbitrary *k*.

### 3.1. The *k*-RF measure for 1-labeled unrooted trees

Suppose *X* is a finite set and *T* is a 1-labeled tree on *X*. Each $e=\left(u,v\right)\in E\left(T\right)$ induces a pair of label subsets on *X*:

(1) $$P_{T}\left(e\right)=\left\{L\left(B_{e}\left(u\right)\right),L\left(B_{e}\left(v\right)\right)\right\},$$

$$B_{e}\left(u\right)=\left\{w|d_{T}\left(w,u\right)<d_{T}\left(w,v\right)\right\},$$

(2) $$B_{e}\left(v\right)=\left\{w|d_{T}\left(w,v\right)<d_{T}\left(w,u\right)\right\}.$$

We further define:

(3) $$P\left(T\right)=\left\{P_{T}\left(e\right)|e\in E\left(T\right)\right\}.$$

The RF distance of two 1-labeled trees *S* and *T* is defined as:

(4) $$d_{RF}\left(S,T\right)=\left|P\left(S\right)\Delta P\left(T\right)\right|.$$

**FIG. 1.**
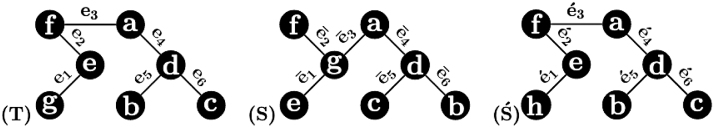
Three 1-labeled trees in Example 1 to illustrate that the Robinson–Foulds distance exhibits a heavy penalty against trees with different labels. Although *T* and S′ is only different in labeling one node, the RF distance is 4 for *S* and *T*, but 12 for S′ and *T*. RF, Robinson–Foulds.

**Example 1.** *For the three 1-labeled trees in* Figure 1*,* $d_{RF}\left(S,T\right)=4$ *but* $d_{RF}\left(S\prime ,T\right)=12$ *although T and* S′ *have the same topology and their label sets are different in only one label.*

The above example illustrates that if two 1-labeled trees have different label sets, their local similarity are not captured by the RF distance. One of the widely used measures for the comparison of sets is the Jaccard distance. It is defined as a fraction whose numerator is the size of the symmetric difference of two sets and whose denominator is the size of their union. Two 1-labeled trees are identical if and only if they have the same set of edges with the understanding that each node is uniquely determined by its label. Hence, we aim to use $\left|E\left(S\right)\Delta E\left(T\right)\right|$ and its generalization to measure the dissimilarity between 1-labeled trees *S* and *T*.

Let k≥0 be an integer and let *T* be a 1-labeled tree. Each edge $e=\left(u,v\right)$ induces the following pair of subsets of labels:

(5) $$\begin{array}{cc} & P_{T}\left(e,k\right)=\left\{L\left(B_{e}\left(u,k\right)\right),L\left(B_{e}\left(v,k\right)\right)\right\},\\ & B_{e}\left(x,k\right)=\left\{w\in B_{e}\left(x\right)|d_{T}\left(w,x\right)\leq k\right\},x=u,v. \end{array}$$

Clearly, $B_{e}\left(u,\infty \right)=B_{e}\left(u\right)$ and $B_{e}\left(u,0\right)=\left\{u\right\}$. We further define:

(6) $$P_{k}\left(T\right)=\left\{P_{T}\left(e,k\right)|e\in E\left(T\right)\right\}.$$

**Definition 1.** *Let k* ≥ 0 *and let S and T be two 1-labeled trees. The k-RF dissimilarity score of S and T is defined as:*

(7) $$d_{k-RF}\left(S,T\right)=\left|P_{k}\left(S\right)\Delta P_{k}\left(T\right)\right|.$$

**Example 2.** *Continuing with Example 1, we have $d_{1-RF}\left(S\prime ,T\right)=4$, as* $P_{T}\left(e_{i},1\right)$ *for* 1≤i≤6 *are:*

$$\begin{array}{cc} & \left\{\left\{g\right\},\left\{e,f\right\}\right\},\left\{\left\{e,g\right\},\left\{a,f\right\}\right\},\left\{\left\{e,f\right\},\left\{a,d\right\}\right\},\\ & \left\{\left\{a,f\right\},\left\{b,c,d\right\}\right\},\left\{\left\{b\right\},\left\{a,c,d\right\}\right\},\left\{\left\{c\right\},\left\{a,b,d\right\}\right\}, \end{array}$$

*respectively, and* $P_{S\prime }\left(e\prime_{i},1\right)$ *for* 1≤i≤6 *are:*

$$\begin{array}{cc} & \left\{\left\{h\right\},\left\{e,f\right\}\right\},\left\{\left\{e,h\right\},\left\{a,f\right\}\right\},\left\{\left\{e,f\right\},\left\{a,d\right\}\right\},\\ & \left\{\left\{a,f\right\},\left\{b,c,d\right\}\right\},\left\{\left\{b\right\},\left\{a,c,d\right\}\right\},\left\{\left\{c\right\},\left\{a,b,d\right\}\right\}, \end{array}$$

*respectively. We also have* $d_{1-RF}\left(S,T\right)=8$. *Thus, 1-RF captures the difference of the trees better than the RF distance.*

### 3.2. The *k*-RF measure for 1-labeled rooted trees

Let *k* ≥ 0 be an integer and let *T* be a 1-labeled rooted tree. For a node $w\in V\left(T\right)$, we define $B_{k}\left(w\right)$ and $D_{k}\left(w\right)$ as:

(8) $$B_{k}\left(w\right)=\left\{x\in V\left(T\right)|\exists y\in A_{T}\left(w\right)\cup \left\{w\right\}:d\left(y,w\right)+d\left(y,x\right)\leq k\right\},$$

(9) $$D_{k}\left(w\right)=\left\{w\right\}\cup \left\{x\in D_{T}\left(w\right)|d\left(w,x\right)\leq k\right\}.$$

For each $e=\left(u,v\right)\in E\left(T\right)$, we define:

(10) $$P_{T}\left(e,k\right)=\left(L\left(D_{k}\left(v\right)\right),L\left(B_{k}\left(u\right)\setminus D_{k}\left(v\right)\right)\right),$$

(11) $$P_{k}\left(T\right)=\left\{P_{T}\left(e,k\right)|e\in E\left(T\right)\right\}.$$

**Definition 2.** *Let k ≥ 0. The k-RF dissimilarity between two 1-labeled rooted trees S and T is defined as:*

(12) $$d_{k-RF}\left(S,T\right)=\left|P_{k}\left(S\right)\Delta P_{k}\left(T\right)\right|.$$

**FIG. 2.**
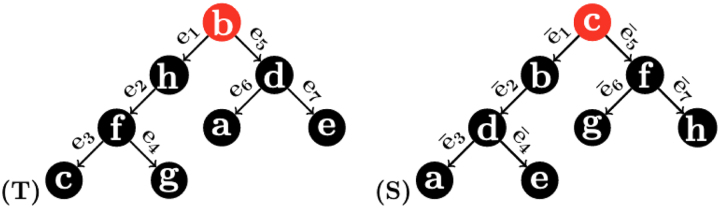
Two 1-labeled rooted trees used to illustrate the 1-RF in Example 3.

**Example 3.** *Consider the two 1-labeled rooted trees S and T in* Figure 2*. We have:*

$$\begin{array}{cc} & P_{T}\left(e_{1},1\right)=\left(\left\{f,h\right\},\left\{b,d\right\}\right),P_{T}\left(e_{2},1\right)=\left(\left\{c,f,g\right\},\left\{b,h\right\}\right),\\ & P_{T}\left(e_{3},1\right)=\left(\left\{c\right\},\left\{f,g,h\right\}\right),P_{T}\left(e_{4},1\right)=\left(\left\{g\right\},\left\{c,f,h\right\}\right)=P_{S}\left({ē}_{6},1\right),\\ & P_{T}\left(e_{5},1\right)=\left(\left\{a,d,e\right\},\left\{b,h\right\}\right),P_{T}\left(e_{6},1\right)=\left(\left\{a\right\},\left\{b,d,e\right\}\right)=P_{S}\left({ē}_{3},1\right),\\ & P_{T}\left(e_{7},1\right)=\left(\left\{e\right\},\left\{a,b,d\right\}\right)=P_{S}\left({ē}_{4},1\right)\\ & P_{S}\left({ē}_{1},1\right)=\left(\left\{b,d\right\},\left\{c,f\right\}\right),P_{S}\left({ē}_{2},1\right)=\left(\left\{a,d,e\right\},\left\{b,c\right\}\right),\\ & P_{S}\left({ē}_{5},1\right)=\left(\left\{f,g,h\right\},\left\{b,c\right\}\right),P_{S}\left({ē}_{7},1\right)=\left(\left\{h\right\},\left\{c,f,g\right\}\right). \end{array},$$

*This implies that* $d_{1-RF}\left(S,T\right)=8$.

## 4. CHARACTERIZATION OF *k*-RF FOR 1-LABELED TREES

To assess k-RF measures, we initially give their mathematical properties. Subsequently, we present experimental findings regarding their frequency distribution.

### 4.1. Mathematical properties

**Proposition 1.** *Let S and T be two 1-labeled trees.*

(a) *For any k ≥ 1*, $d_{k-RF}\left(S,T\right)=\left|E\left(S\right)\right|+\left|E\left(T\right)\right|$ *if S and T share at most 2 labels and there are at least two edges in either S or T*.
(b) *Assume that* $L\left(S\right)\neq L\left(T\right)$. *For* $k<min\left\{diam\left(T\right),diam\left(S\right)\right\}$, $k+1\leq d_{k-RF}\left(S,T\right)\leq \left|E\left(S\right)\right|+\left|E\left(T\right)\right|$. *In addition, the second inequality become equality if* $k\leq min\left\{diam\left(T\right),diam\left(S\right)\right\}$ *and* $\left|L\left(S\right)\right|=\left|L\left(T\right)\right|$.
(c) *Renaming each node with its label, we have* $d_{0-RF}\left(S,T\right)=\left|E\left(S\right)\Delta E\left(T\right)\right|$.
(d) *If* $k\geq max\left\{diam\left(S\right),diam\left(T\right)\right\}-1$, *then* $d_{k-RF}\left(S,T\right)=d_{RF}\left(S,T\right)$.

*Proof.* (a) Note that if *k* ≥ 1 and $\left|E\left(T\right)\right|\geq 2$, each $P_{T}\left(e,k\right)$ involves at least three labels. If *L*(*S*) and *L*(*T*) have only two common elements, $P_{T}\left(e,k\right)\neq P_{S}\left(e\prime ,k\right)$ for every $e\in E\left(T\right)$ and $e\prime \in E\left(S\right)$. Thus, we have $P_{k}\left(S\right)\cap P_{k}\left(T\right)=\emptyset$, implying that $d_{k-RF}\left(S,T\right)=|P_{k}\left(S\right)\Delta P_{k}\left(T\right)\left|=\right|P_{k}\left(T\right)\left|+\right|P_{k}\left(S\right)=\left|E\left(S\right)\right|+\left|E\left(T\right)\right|.$

(b) The second inequality follows from that $d_{k-RF}\left(S,T\right)=\left|P_{k}\left(S\right)\Delta P_{k}\left(T\right)\left|\leq \right|P_{k}\left(T\right)\left|+\right|P_{k}\left(S\right)\right|$ and $\left|P_{k}\left(X\right)\right|=\left|E\left(X\right)\right|$ for X=S,T. We prove the first inequality as follows.

Let $k<min\left\{diam\left(T\right),diam\left(S\right)\right\}$. Since *S* and *T* are 1-labeled, we identify a node with its label in the trees. Without loss of generality, we may assume $v\in V\left(T\right)\setminus V\left(S\right)$. Define $N_{T}^{\left(k\right)}\left(v\right)=\left\{u|d_{T}\left(u,v\right)\leq k\right\}$.

If $N_{T}^{\left(k\right)}\left(v\right)=V\left(T\right)$, then, $\left|N_{T}^{\left(k\right)}\left(v\right)\right|=\left|V\left(T\right)\right|\geq diam\left(T\right)+1\geq k+2$, as $k<diam\left(T\right)$. This also implies that for every $\left(x,y\right)\in E\left(T\right)$, $d_{T}\left(v,x\right)\leq k$ and $d_{T}\left(v,y\right)\leq k$.

If $N_{T}^{\left(k\right)}\left(v\right)\neq V\left(T\right)$, there exists at least a node *w* that is *k* + 1 or more distance away from *v*. Since *T* is connected, we let $P\left(v,w\right)$ be a path from *v* and *w* with the smallest length. Clearly, the first k+1 nodes in $P\left(v,w\right)$ (including *v*) are all in $N_{T}^{\left(k\right)}\left(v\right)$, that is, at least one end of the first *k* + 1 edges of $P\left(v,w\right)$ are found in $N_{T}^{\left(k\right)}\left(v\right)$.

In summary, we have proved that there are at least *k* + 1 edges (*x,y*) such that either $d_{T}\left(v,x\right)\leq k$ or $d_{T}\left(v,y\right)\leq k$. For each of these edges *e*, *v* appears in at least one label subset of $P_{T}\left(e,k\right)$ and thus $P_{T}\left(e,k\right)\notin P_{k}\left(S\right)$. Therefore, $d_{k-RF}\left(S,T\right)\geq \left|P_{k}\left(T\right)\setminus P_{k}\left(S\right)\right|\geq k+1$.

If $\left|L\left(S\right)\right|=\left|L\left(T\right)\right|$ and $k\geq min\left\{diam\left(T\right),diam\left(S\right)\right\}$, then, $N_{T}^{\left(k\right)}\left(v\right)=V\left(T\right)$. Therefore, the induced pair $P_{T}\left(e,k\right)$ contains *v* for every edge *e* of *T*. On the contrary, the induced pair $P_{S}\left(e,k\right)$ does not contain *v* for each edge *e* of *S*. Thus, $P_{k}\left(S\right)\cap P_{k}\left(T\right)=\emptyset$ and $d_{k-RF}\left(S,T\right)=\left|P_{k}\left(S\right)\left|+\right|P_{k}\left(T\right)\right|=\left|E\left(S\right)\right|+\left|E\left(T\right)\right|$.

(c) Note that we may represent each node of a 1-labeled tree with its unique label. As a result, $P_{T}\left(e,0\right)=e$ and $P_{S}\left(ē,0\right)=e$ for $e\in E\left(T\right)$ and $ē\in E\left(S\right)$. Thus, (c) follows.

(d) It follows from the definition of the *k*-RF.

**Lemma 1.** *Let k ≥ 0 be an integer. k*-*RF satisfies the non-negativity, symmetry and triangle inequality conditions.*

*Proof.* Let *k* ≥ 0. The non-negativity and symmetry conditions are trivial. The triangle inequality $d_{k-RF}\left(T_{1},T_{2}\right)\leq d_{k-RF}\left(T_{1},T_{3}\right)+d_{k-RF}\left(T_{3},T_{2}\right)$ is derived from the inequality $P_{k}\left(T_{1}\right)\Delta P_{k}\left(T_{2}\right)\subseteq \left(P_{k}\left(T_{1}\right)\Delta P_{k}\left(T_{3}\right)\right)\cup \left(P_{k}\left(T_{3}\right)\Delta P_{k}\left(T_{2}\right)\right)$ for any three 1-labeled trees $T_{1},T_{2},T_{3}$.

**Remark 1.** *Proposition 1 and Lemma 1 can be proved in the same manner for 1-labeled rooted trees.*

**Proposition 2.** *The 0-RF is a metric on the space of all 1-labeled rooted trees.*

*Proof.* Let *S* and *T* be two 1-labeled rooted trees. By Remark 1, it is enough to show that *S* and *T* are identical if $d_{0-RF}\left(S,T\right)=0$. By identifying a node with its label in *S* and *T*, we obtain that $P_{0}\left(S\right)=E\left(S\right)$ and $P_{0}\left(T\right)=E\left(T\right)$. If $d_{0-RF}\left(S,T\right)=0$, $\left|E\left(T\right)\Delta E\left(S\right)\right|=0$ and thus $E\left(T\right)=E\left(S\right)$, that is, *S* and *T* are identical.

**Lemma 2.** *Let T be a 1-labeled rooted tree with at least two nodes and* ℒ *be a subset of Leaf (T)*. *Define T′ to be the subtree obtained by the removal of all the leaves of* ℒ. *Then, for any k*,

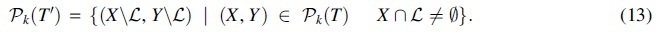

*Proof.* Since *T* is 1-labeled, we identify a node of *T* with its label in the following discussion. With this convention, for any subset *S* of nodes, $L\left(S\right)=S$.

Let $Ē\left(T\right)$ denote the subset of edges incident to a leaf of ℒ, that is, $Ē\left(T\right)=\left\{\left(x,y\right)\in E\left(T\right)|y\in ℒ\right\}$. Then,

$$V\left(T\right)=V\left(T\prime \right)⊎ℒ,E\left(T\right)=E\left(T\prime \right)⊎Ē\left(T\right).$$

If $\left(u,v\right)\in Ē\left(T\right)$, $v\in ℒ\subseteq Leaf\left(T\right)$ and thus $D_{k}\left(v\right)=\left\{v\right\}\subseteq ℒ.$

For an edge $e=\left(u,v\right)\in E\left(T\prime \right)$, $P_{T}\left(e,k\right)=\left(D_{k}\left(v\right),B_{k}\left(u\right)\setminus D_{k}\left(v\right)\right)$. By Equations (8) and (9),

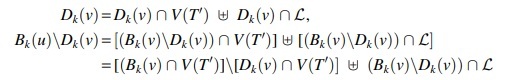

If $\left(u,v\right)\in E\left(T\prime \right)$, $D_{k}\left(v\right)\setminus ℒ=D_{k}\left(v\right)\cap V\left(T\prime \right)\neq \emptyset$ and $\left(B_{k}\left(v\right)\setminus D_{k}\left(v\right)\right)\setminus ℒ=\left(B_{k}\left(v\right)\cap V\left(T\prime \right)\{]\}\setminus \{[\}D_{k}\left(v\right)\cap V\left(T\prime \right)\right].$ Therefore, $\left(D_{k}\left(v\right)\setminus ℒ,\left(B_{k}\left(v\right)\setminus D_{k}\left(v\right)\right)\setminus ℒ\right)=P_{T\prime }\left(e,k\right).$

This has proved Equation (13).

**Proposition 3.** *Let k* ≥ *1 be an integer. k-RF is a metric in the space of all 1-labeled rooted trees.*

*Proof.* Let *S* and *T* be two 1-labeled rooted trees. By Remark 1, it is enough to show that $d_{k-RF}\left(S,T\right)=0$ (equivalently, $P_{k}\left(T\right)=P_{k}\left(S\right)$) implies that *S* and *T* are identical. To this end, we prove that *E*(*T*) can be uniquely determined by $P_{k}\left(T\right)$ using mathematical induction.

Since $\left|E\left(T\right)\right|=\left|P_{k}\left(T\right)\right|$, *T* is a single node if and only if *E*(*T*) is empty if and only $P_{k}\left(T\right)$ is empty. Therefore, the single-node tree is uniquely determined by $P_{k}\left(T\right)$.

Assume *S* is uniquely determined by $P_{k}\left(S\right)$ for arbitrary 1-labeled tree *S* such that $\left|V\left(S\right)\right|<k$. Consider a 1-labeled tree *T* such that $\left|V\left(S\right)\right|=k$.

For a leaf $v\in Leaf\left(T\right)$, there is a unique edge $e=\left(u,v\right)$ entering *v*. Note that *k* ≥ 1. Since $D_{k}\left(v\right)=\left\{v\right\}$ if and only if *v* is a leaf, we can identify *v* from $P_{T}\left(e,k\right)=\left(P_{1},P_{2}\right)\in P_{k}\left(T\right)$ such that $P_{1}=\left\{v\right\}.$.

For $v\in V\left(T\right)\setminus Leaf\left(T\right)$, there is a unique edge $e=\left(u,v\right)$ entering *v*. Since *k* ≥ 1, the children of *v* are all a leaf if and only if $D_{k}\left(v\right)=\left\{v\right\}\cup C_{T}\left(u\right)$ if and only if $D_{K}\left(v\right)\setminus Leaf\left(T\right)=\left\{v\right\}$. Therefore, we can identify *v* whose children are all leaves from the ordered pairs $\left(P_{1},P_{2}\right)\in P_{k}\left(T\right)$ such that $P_{1}\setminus Leaf\left(T\right)$ contains only *v*.

Let *V*′ be the set of all nodes whose children are just leaves and $D_{T}\left(V\prime \right)=\cup_{x\in V\prime }C_{T}\left(x\right)$. Since V′ is nonempty, $D_{T}\left(V\prime \right)\neq \emptyset$. Define $E\prime \left(T\right)=\left\{\left(x,y\right)\in E\left(T\right)|x\in V\prime ,y\in D_{T}\left(V\prime \right)\right\}$.

For the tree *T*′ obtained from *T* by the removal of the leaves of $D_{T}\left(V\prime \right)$, $\left|V\left(T\prime \right)\right|=\left|V\left(S\right)\right|-\left|D_{T}\left(V\prime \right)\right|<k.$ By Equation (13), $P_{k}\left(T\prime \right)$ can be efficiently constructed from $P_{k}\left(T\right)$. By the induction hypothesis, $E\left(T\prime \right)$ is uniquely determined by $P_{k}\left(T\prime \right)$. As a result, $E\left(T\right)=E\left(T\prime \right)\cup E\prime \left(T\right)$ is determined.

This concludes the proof.

**Corollary 1.** *Let k* ≥ 0. *The k*-*RF is a metric in the space of all 1-labeled trees.*

*Proof.* If *k* = 0, the statement follows from the same proof as for Proposition 2. Now, let *S* and *T* be two 1-labeled trees and *k* ≥ 1. By Lemma 1, it is enough to show that if $d_{k-RF}\left(S,T\right)=0$ (equivalently, $P_{k}\left(T\right)=P_{k}\left(S\right)$), then *S* and *T*. This can be proved in a manner similar to Proposition 3.

**Lemma 3.** *Let k ≥ 0 and let T be a 1-labeled rooted tree with n nodes. All subsets* $D_{i}\left(w\right)=\left\{w\right\}\cup \left\{x\in D_{T}\left(w\right)|d\left(w,x\right)\leq i\right\}$ *and* $L\left(D_{i}\left(w\right)\right)$ *for all nodes w and i ≤ k can be computed in at most* $2\left(k+1\right)n$ *set operations.*

*Proof.* Since *T* is 1-labeled, we can identify a node of *T* with its label. In this way, $D_{i}\left(w\right)=L\left(D_{i}\left(w\right)\right)$ for all nodes *w* and *i ≤ k*. By ordering the *n* labels, we represent each subset of labels (and each subset of nodes) as a *n*-bit 0–1 string, where the *i*-th bit is 1 if and only if the *i*-th label (node) is in the subset.

The statement is obvious in the case *k = 0*, since $D_{0}\left(w\right)=\left\{w\right\}$ and, clearly, all the $D_{0}\left(w\right)$ for $w\in V\left(T\right)$ can be computed in at most 2*n* set operations. We assume *k > 0* and prove the statement by induction as follows.

Assume that all the $D_{k-1}\left(w\right)$ for $w\in V\left(T\right)$ have been computed in at most 2*kn* set operations. Assume *w* has *d_w_* children $u_{1},u_{2},\ldots ,u_{d\left(w\right)}$. Then,

$$D_{k}\left(w\right)=\left\{w\right\}\cup \left(\cup_{i=1}^{d_{w}}D_{k-1}\left(u_{i}\right)\right)$$

This implies that $D_{k}\left(w\right)$ for all *w* can be computed from all $D_{k-1}\left(w\right)$ using 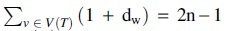 set operations. In total, we can compute all subsets 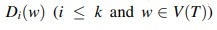 in at most $2n-1+2kn\leq 2\left(k+1\right)n$ set operations.

**Lemma 4.** *Let k ≥ 0 and T be a 1-labeled rooted tree with n nodes. Using* $L\left(D_{i}\left(w\right)\right)$ *for* $w\in V\left(T\right),0\leq i\leq k$, *we can compute* $L\left(B_{k}\left(w\right)\right)$ *for all w in* $O\left(kn\right)$ *set operations, where* $B_{k}\left(w\right)$ *is defined in Equation (8).*

*Proof.* Since *T* is a 1-labeled rooted tree, we identify a node with its label. In this way, we just need to show that $B_{k}\left(w\right)$ for all nodes *w* can be computed in $O\left(kn\right)$ set operations.

Let *r* be the root of *T*. For any node $w\in V\left(T\right)$, let the unique path from *r* to *w* be

$$w_{0}=r,w_{1},\ldots ,w_{t}=w.$$

Then, we have that

$$B_{k}\left(w_{t}\right)=\cup_{i=0}^{min\left(k,t\right)}D_{k-i}\left(w_{t-i}\right).$$

Given the subsets $D_{i}\left(u\right)$ for all i≤k and $u\in V\left(T\right)$, the above formula implies that $B_{k}\left(w_{t}\right)$ can be computed in at most *k* set operations. In total, we can compute all $B_{k}\left(w_{t}\right)$ for all $w\in V\left(T\right)$ in $O\left(kn\right)$ set operations.

**Proposition 4.** *Let S and T be two 1-labeled trees with n nodes and k ≥ 0. Then,* $d_{k-RF}\left(S,T\right)$ *can be computed in* $O\left(kn^{2}\right)$ *time.*

*Proof.* We first consider the rooted tree case. Let *S* and *T* be two 1-labeled rooted trees with *n* nodes. Without loss of generality, we may assume that *S* and *T* are labeled on the same set *L*, with $\left|L\right|=n$. (Otherwise, we can consider them labeled on $L=L\left(S\right)\cup L\left(T\right)$, with $n\leq \left|L\right|\leq 2n$.) By Lemma 3 and Lemma 4, we can compute $P_{X}\left(e,k\right)$ for all $e\in E\left(X\right)$ in $O\left(kn\right)$ set operations for *X* = *S,T*. Since each edge induces an ordered pair of label subsets and we represent each label subset using a *n*-bit string, we consider $P_{X}\left(e,k\right)$ as a 2*n*-bit string. In this way, we sort all the edge-induced pairs of label subsets for each tree in $O\left(n^{2}\right)$ time by radix sort (i.e., indexing) and then compute the symmetric difference of the two set of edge-induced pairs in $O\left(n^{2}\right)$ time. This concludes the proof.

In the unrooted case, we first root the trees at a leaf. In this way, we can compute all the edge-induced pairs of label subsets in the derived rooted trees in $O\left(kn^{2}\right)$ time. Since the edges induce unordered pairs of label subsets in the original trees, we rearrange the two label subsets obtained for an edge in such a way that the smallest label in the first subset is smaller than every label in the second one. After the rearrangement, we can radix-sort the edge-induced pairs and compute the *k*-RF score in $O\left(n^{2}\right)$ time.

### 4.2. Distribution of *k*-RF scores

We examined the distribution of the *k*-RF dissimilarity scores for 1-labeled unrooted and rooted trees with the same label set and with different label sets.

**FIG. 3.**
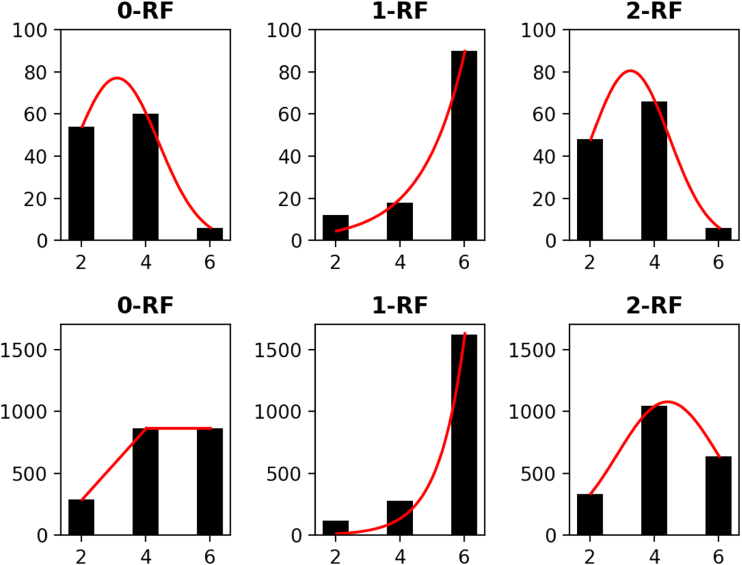
The frequency distributions of all pairwise *k*-RF scores in the space of 1-labeled unrooted (top row) and rooted (bottom row) 4-node trees for k=0,1,2. In the bar-charts, the *x*axis represents *k*-RF scores and the *y*-axis represents the number of tree pairs with a specific *k*-RF score.

**FIG. 4.**
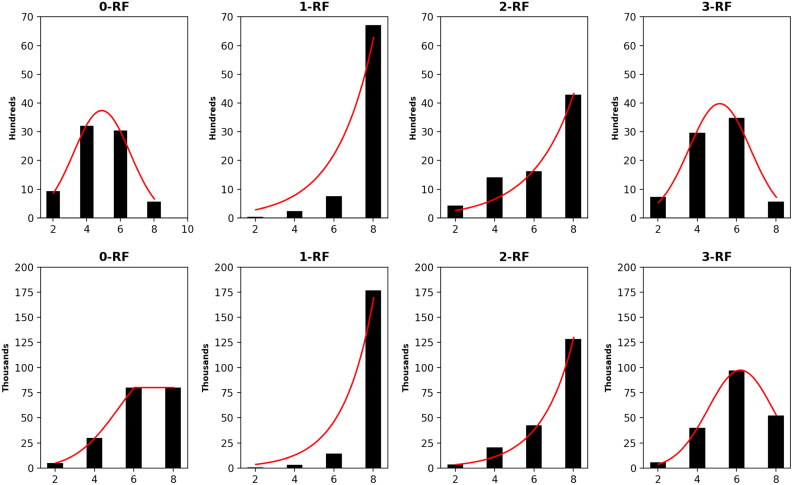
The frequency distributions of all pairwise *k-*RF scores in the space of 1-labeled unrooted (top row) and rooted (bottom row) 5-node trees, where *k* ≤ 3. In the bar-charts, the *x*-axis represents *k*-RF scores and the *y*-axis represents the number of tree pairs with a specific *k*-RF score.

**FIG. 5.**
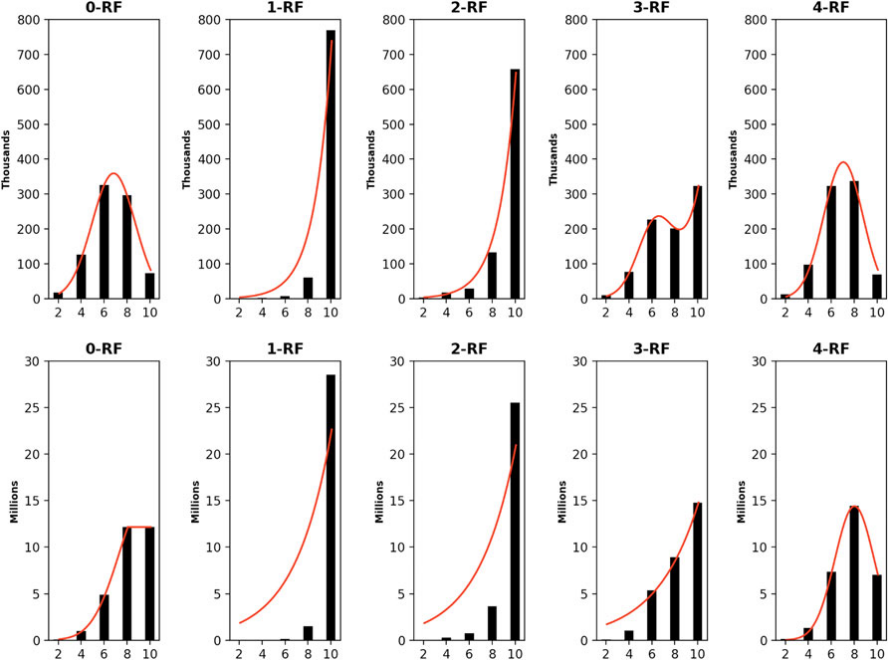
The frequency distributions of all pairwise *k*-RF scores in the space of 1-labeled unrooted (top row) and rooted (bottom row) 6-node trees for *k* ≤ 4. In each bar-chart, the *x*-axis represents *k*-RF scores and the *y*-axis represents the number of tree pairs whose *k*-RF equals a given score.

**FIG. 6.**
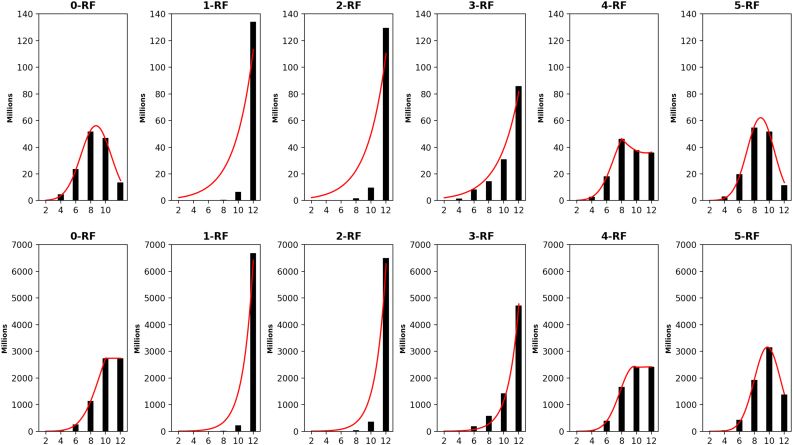
The frequency distributions of all pairwise *k-*RF scores in the space of 1-labeled unrooted (top row) and rooted (bottom row) 7-node trees for *k* ≤ 5. In each bar-chart, the *x*-axis represents *k*-RF scores and the *y*-axis represents the number of tree pairs whose *k*-RF equals a given score.

The distribution of the frequency of the pairwise *k*-RF scores in the space of *n*-node 1-labeled unrooted and rooted trees for *n* from 4 to 7 are presented in Figures 3 to 6, respectively. For each *n*, it suffices to consider k=0,…,n−2. Recall that (*n* − 2) -RF is actually the RF distance. The frequency distribution for the RF distance in the space of phylogenetic trees is known to be Poisson (Steel and Penny, 1993). It seems also true that the pairwise 0-RF and (*n* − 2) -RF scores have a Poisson distribution in the space of *n*-node 1-labeled unrooted and rooted trees. However, the distribution of the pairwise *k*-RF scores is unlikely Poisson when k=1,2,3 and k≠n−2.

We examined 1,679,616 (respectively, 60,466,176) pairs of 6-node 1-labeled unrooted (respectively, rooted) trees such that the trees in each pair have *c* common labels, with c=3,4,5. Table 1 shows that the majority of pairs have a largest dissimilarity score of 10.

**Table 1.** Number of Pairs of 1-Labeled 6-Node Unrooted (Top) and Rooted (Bottom) Trees That Have *c* Labels in Common and Have 1-Robinson–Foulds Score *d* for c=3,4,5 and d=2,4,6,8,10

1-RF	2	4	6	8	10
3	0	0	0	3072	1,676,544
4	0	0	432	16,800	1,662,384
5	0	340	3720	53,100	1,622,456

1-RF	2	4	6	8	10
3	0	0	0	79,872	60,386,304
4	0	0	7776	419,136	60,039,264
5	0	4080	65,760	1,310,880	59,085,456

RF, Robinson–Foulds.

## 5. A GENERALIZATION TO MULTISET-LABELED TREES

In this section, we extend the measures introduced in Section 3 to multiset-labeled unrooted and rooted trees.

### 5.1. Multisets and their operations

A multiset is a collection of elements in which an element *x* can occur one or more times (Jűrgensen, 2020). The set of all distinct elements appearing in a multiset *A* is denoted by Supp (*A*). In this study, we simply represent *A* by the monomial $x_{1}^{m_{A}\left(x_{1}\right)}\ldots x_{n}^{m_{A}\left(x_{n}\right)}$ if $Supp\left(A\right)=\left\{x_{1},x_{2},\cdots ,x_{n}\right\}$, where $x_{i}^{1}$ is simplified to *x_i_* for each *i*.

Let *A* and *B* be two multisets. We say *A* is a sub-multiset of *B*, denoted by $A\subseteq_{m}B$, if for every $x\in Supp\left(A\right)$, $m_{A}\left(x\right)\leq m_{B}\left(x\right)$. In addition, we say that A=B if $A\subseteq_{m}B$, and $B\subseteq_{m}A$. Furthermore, the union, sum, intersection, difference, and symmetric difference of *A* and *B* are, respectively, defined as follows:

- $A\cup_{m}B=\left\{x^{max\left\{m_{A}\left(x\right),m_{B}\left(x\right)\right\}}|x\in Supp\left(A\right)\cup Supp\left(B\right)\right\}$;
- $A{⊎}_{m}B=\left\{x^{m_{A}\left(x\right)+m_{B}\left(x\right)}|x\in Supp\left(A\right)\cup Supp\left(B\right)\right\}$;
- $A\cap_{m}B=\left\{x^{min\left\{m_{A}\left(x\right),m_{B}\left(x\right)\right\}}|x\in Supp\left(A\right)\cap Supp\left(B\right)\right\}$;
- $A\setminus_{m}B=\left\{x^{m_{A}\left(x\right)-m_{B}\left(x\right)}|x\in Supp\left(A\right):m_{A}\left(x\right)>m_{B}\left(x\right)\right\}$;
- $A\Delta_{m}B=\left(A\cup_{m}B\right)\setminus_{m}\left(A\cap_{m}B\right)$;

where $m_{X}\left(x\right)$ is defined as 0 if $x\notin Supp\left(X\right)$ for X=A,B.

Let *L* be a set and $P_{m}\left(L\right)$ be the set of all sub-multisets on *L*. A tree *T* is labeled with the sub-multisets of *L* if *T* is equipped with a function $\ell :V\left(T\right)\to P_{m}\left(L\right)$ such that $\cup_{v\in V\left(T\right)}Supp\left(\ell \left(v\right)\right)=L$ and $\ell \left(v\right)\neq \emptyset$, for every $v\in V\left(T\right)$. We call such a tree as a multiset-labeled tree. For $C\subseteq V\left(T\right)$ and x∈L, we define $L_{m}\left(C\right)$ and $m_{T}\left(x\right)$ as follows:

(14) $$L_{m}\left(C\right)={⊎}_{v\in C}\ell \left(v\right);$$

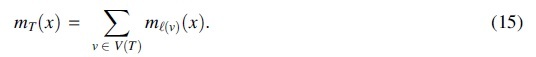


### 5.2. The *k*-RF for multiset-labeled trees

Let *T* be a multiset-labeled tree. Then, each edge $e=\left(u,v\right)$ of *T* induces a pair of multisets

(16) $$P_{T}\left(e\right)=\left\{L_{m}\left(B_{e}\left(u\right)\right),L_{m}\left(B_{e}\left(v\right)\right)\right\},$$

where $L_{m}\left(\right)$ is defined in Equation (14), and $B_{e}\left(u\right)$ in Equation (2). Note that Equation (16) is obtained from Equation (1) by replacing L() with $L_{m}\left(\right)$.

**FIG. 7.**
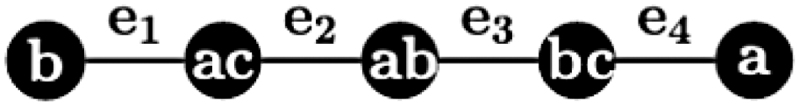
Two multiset-labeled trees used to show that different edges can give the same label multi-subset pair. Here, $P_{T}\left(e_{2}\right)=P_{T}\left(e_{3}\right)=\left\{abc,a^{2}b^{2}c\right\}$.

**Remark 2.** *In a multiset-labeled tree T, two edges may induce the same multi-set pair as shown in* Figure 7*. Hence,* $P\left(T\right)$ *in Equation (3) is a multiset in general.*

We use Equations (16), (3), and (4) to define the RF-distance for multiset-labeled trees by replacing Δ with $\Delta_{m}$ in Equation (4).

Let *k* ≥ 0. We use Equations (5), (6), and (7) to define the *k*-RF for multiset-labeled trees by replacing L() with $L_{m}\left(\right)$ in Equation (5) and replacing Δ with $\Delta_{m}$ in Equation (7).

**FIG. 8.**
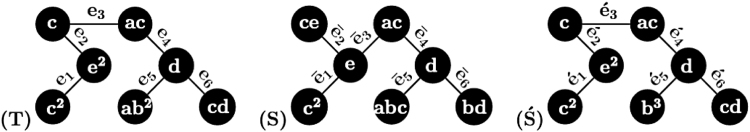
Three multiset-labeled trees in Example 4.

**Example 4.** *Consider the multiset-labeled trees S, S′, and T in* Figure 8*.* $P_{k}\left(T\right),P_{k}\left(S\right)$ *and* $P_{k}\left(S\prime \right)$ *for* k=0,1,∞ *are summarized in* Table 2*. We obtain:*

**Table 2.** Edge-Induced Unordered Pairs of Multisets in the Three Trees in Figure 8 for k=0,1,∞

Tree	$P_{0}\left(\right)$	$P_{1}\left(\right)$	$P_{\infty }\left(\right)$
*T*	$\left\{c^{2},e^{2}\right\}$	$\left\{c^{2},ce^{2}\right\}$	$\left\{a^{2}b^{2}c^{3}d^{2}e^{2},c^{2}\right\}$
	$\left\{c,e^{2}\right\}$	$\left\{ab^{2}cd^{2},ac^{2}\right\}$	$\left\{a^{2}b^{2}c^{3}d^{2},c^{2}e^{2}\right\}$
	$\left\{ac,c\right\}$	$\left\{ac^{2},c^{2}e^{2}\right\}$	$\left\{a^{2}b^{2}c^{2}d^{2},c^{3}e^{2}\right\}$
	$\left\{ac,d\right\}$	$\left\{ab^{2},ac^{2}d^{2}\right\}$	$\left\{ab^{2}cd^{2},ac^{4}e^{2}\right\}$
	$\left\{ab^{2},d\right\}$	$\left\{acd,ce^{2}\right\}$	$\left\{ab^{2},ac^{5}d^{2}e^{2}\right\}$
	$\left\{cd,d\right\}$	$\left\{a^{2}b^{2}cd,cd\right\}$	$\left\{a^{2}b^{2}c^{4}de^{2},cd\right\}$
*S*	$\left\{c^{2},e\right\}$	$\left\{ac^{2}e^{2},c^{2}\right\}$	$\left\{a^{2}b^{2}c^{3}d^{2}e^{2},c^{2}\right\}$
	$\left\{ce,e\right\}$	$\left\{a^{2}bc^{2}d,bd\right\}$	$\left\{a^{2}b^{2}c^{2}d^{2},c^{3}e^{2}\right\}$
	$\left\{ac,e\right\}$	$\left\{ab^{2}cd^{2},ace\right\}$	$\left\{ab^{2}cd^{2},ac^{4}e^{2}\right\}$
	$\left\{ac,d\right\}$	$\left\{ac^{3}e,ce\right\}$	$\left\{a^{2}b^{2}c^{4}d^{2}e,ce\right\}$
	$\left\{abc,d\right\}$	$\left\{acd,c^{3}e^{2}\right\}$	$\left\{abc,abc^{4}d^{2}e^{2}\right\}$
	$\left\{bd,d\right\}$	$\left\{abc,abcd^{2}\right\}$	$\left\{a^{2}bc^{5}de^{2},bd\right\}$
*Ś*	$\left\{c^{2},e^{2}\right\}$	$\left\{c^{2},ce^{2}\right\},$	$\left\{ab^{3}c^{3}d^{2}e^{2},c^{2}\right\}$
	$\left\{c,e^{2}\right\}$	$\left\{ac^{2},c^{2}e^{2}\right\}$	$\left\{ab^{3}c^{3}d^{2},c^{2}e^{2}\right\}$
	$\left\{ac,c\right\}$	$\left\{acd,ce^{2}\right\}$	$\left\{ab^{3}c^{2}d^{2},c^{3}e^{2}\right\}$
	$\left\{ac,d\right\}$	$\left\{ac^{2}d^{2},b^{3}\right\}$	$\left\{ac^{4}e^{2},b^{3}cd^{2}\right\}$
	$\left\{b^{3},d\right\}$	$\left\{ac^{2},b^{3}cd^{2}\right\}$	$\left\{ac^{5}e^{2}d^{2},b^{3}\right\}$
	$\left\{cd,d\right\}$	$\left\{ab^{3}cd,cd\right\}$	$\left\{ab^{3}c^{4}e^{2}d,cd\right\}$

$$\begin{array}{cc} & d_{0-RF}\left(T,S\prime \right)=2;d_{1-RF}\left(T,S\prime \right)=6;d_{RF}\left(T,S\prime \right)=12;\\ & d_{0-RF}\left(S,S\prime \right)=10;d_{1-RF}\left(S,S\prime \right)=12;d_{RF}\left(S,S\prime \right)=12. \end{array}$$

*It is not hard to see that both $d_{0-RF}\left(T,S\prime \right)$ and $d_{1-RF}\left(T,S\prime \right)$ reflect the local similarity of the two multiset-labeled trees better than* $d_{RF}\left(T,S\prime \right)$.

### 5.3. *k*-RF for multiset-labeled rooted trees

Let *k* ≥ 0 be an integer. We use Equations (10), (11), and (12) to define *k*-RF for multiset-labeled rooted trees by replacing L() with $L_{m}\left(\right)$ in Equation (10) and replacing Δ with $\Delta_{m}$ in Equation (12).

**Proposition 5.** *Let k* ≥ 0 *be an integer. The k-RF satisfies the non-negativity, symmetry, and triangle inequality conditions. Hence, k-RF is a pseudometric for each k in the space of multiset-labeled (rooted) trees.*

*Proof.* The non-negativity and symmetry conditions follow from the definition of the *k*-RF. The triangle inequality condition is proved as follows.

Let *T*_1_, *T*_2_, and *T*_3_ be three multiset-labeled trees. We need to show:

$$d_{k-RF}\left(T_{1},T_{2}\right)\leq d_{k-RF}\left(T_{1},T_{3}\right)+d_{k-RF}\left(T_{3},T_{2}\right).$$

Note that $P_{k}\left(X\right)$ denotes the multiset of edge-induced order pairs of sub-multisets in *X* for $X=T_{1},T_{2},T_{3}$.

If $x^{m\left(x\right)}\in P_{k}\left(T_{1}\right)\Delta_{m}P_{k}\left(T_{2}\right)$, we have either $x^{m\left(x\right)}\in P_{k}\left(T_{1}\right)\setminus_{m}P_{k}\left(T_{2}\right)$ or $x^{m\left(x\right)}\in P_{k}\left(T_{2}\right)\setminus_{m}P_{k}\left(T_{1}\right)$. Assume $x^{m\left(x\right)}\in P_{k}\left(T_{1}\right)\setminus_{m}P_{k}\left(T_{2}\right)$. Then, $m_{P_{k}\left(T_{1}\right)}\left(x\right)>m_{P_{k}\left(T_{2}\right)}\left(x\right)$. If $x\notin Supp\left(P_{k}\left(T_{3}\right)\setminus_{m}P_{k}\left(T_{2}\right)\right)$, we have $m_{P_{k}\left(T_{1}\right)}\left(x\right)>m_{P_{k}\left(T_{2}\right)}\left(x\right)\geq m_{P_{k}\left(T_{3}\right)}\left(x\right)$. This implies that $x\in Supp\left(P_{k}\left(T_{1}\right)\setminus_{m}P_{k}\left(T_{3}\right)\right)$ and $m_{P_{k}\left(T_{1}\right)\setminus_{m}P_{k}\left(T_{3}\right)}\left(x\right)=m_{P_{k}\left(T_{1}\right)}\left(x\right)-m_{P_{k}\left(T_{3}\right)}\left(x\right)\geq m_{P_{k}\left(T_{1}\right)}\left(x\right)-m_{P_{k}\left(T_{2}\right)}\left(x\right)=m\left(x\right).$ Thus, $m\left(x\right)\leq m_{P_{k}\left(T_{1}\right)\Delta_{m}P_{k}\left(T_{3}\right)}\left(x\right)+m_{P_{k}\left(T_{3}\right)\Delta_{m}P_{k}\left(T_{2}\right)}\left(x\right).$

On the contrary, if $x\in Supp\left(P_{k}\left(T_{3}\right)\setminus_{m}P_{k}\left(T_{2}\right)\right)$ and $m_{P_{k}\left(T_{3}\right)}\left(x\right)\geq m_{P_{k}\left(T_{1}\right)}\left(x\right)$, we have:

$$\begin{array}{c} m_{P_{k}\left(T_{3}\right)\setminus_{m}P_{k}\left(T_{2}\right)}\left(x\right)=m_{P_{k}\left(T_{3}\right)}\left(x\right)-m_{P_{k}\left(T_{2}\right)}\left(x\right)\\ \geq m_{P_{k}\left(T_{1}\right)}\left(x\right)-m_{P_{k}\left(T_{2}\right)}\left(x\right)=m\left(x\right). \end{array}$$

If $x\in Supp\left(P_{k}\left(T_{3}\right)\setminus_{m}P_{k}\left(T_{2}\right)\right)$ and $m_{P_{k}\left(T_{3}\right)}\left(x\right)<m_{P_{k}\left(T_{1}\right)}\left(x\right)$, we have $m_{P_{k}\left(T_{1}\right)}\left(x\right)>m_{P_{k}\left(T_{3}\right)}\left(x\right)>m_{P_{k}\left(T_{2}\right)}\left(x\right)$, implying $x\in Supp\left(P_{k}\left(T_{1}\right)\setminus_{m}P_{k}\left(T_{3}\right)\right)$. Thus, we have:

$$\begin{array}{c} m\left(x\right)=m_{P_{k}\left(T_{1}\right)\setminus_{m}P_{k}\left(T_{3}\right)}\left(x\right)+m_{P_{k}\left(T_{3}\right)\setminus_{m}P_{k}\left(T_{2}\right)}\left(x\right)\\ \leq m_{P_{k}\left(T_{1}\right)\Delta_{m}P_{k}\left(T_{3}\right)}\left(x\right)+m_{P_{k}\left(T_{3}\right)\Delta_{m}P_{k}\left(T_{2}\right)}\left(x\right). \end{array}$$

Finally, if $x^{m\left(x\right)}\in P_{k}\left(T_{2}\right)\setminus_{m}P_{k}\left(T_{1}\right)$, we can obtain the same result. In summary, we have:

$$Supp\left(P_{k}\left(T_{1}\right)\Delta_{m}P_{k}\left(T_{2}\right)\right)\subseteq Supp\left(P_{k}\left(T_{1}\right)\Delta_{m}P_{k}\left(T_{3}\right)\right)\cup Supp\left(P_{k}\left(T_{3}\right)\Delta_{m}P_{k}\left(T_{2}\right)\right).$$

In addition, for each $x\in Supp\left(P_{k}\left(T_{1}\right)\Delta_{m}P_{k}\left(T_{2}\right)\right)$, we have:

$$m_{P_{k}\left(T_{1}\right)\Delta_{m}P_{k}\left(T_{2}\right)}\left(x\right)\leq m_{P_{k}\left(T_{1}\right)\Delta_{m}P_{k}\left(T_{3}\right)}\left(x\right)+m_{P_{k}\left(T_{3}\right)\Delta_{m}P_{k}\left(T_{2}\right)}\left(x\right).$$

Therefore, we have:

$$\left|P_{k}\left(T_{1}\right)\Delta_{m}P_{k}\left(T_{2}\right)\left|\leq \right|P_{k}\left(T_{1}\right)\Delta_{m}P_{k}\left(T_{3}\right)\left|+\right|P_{k}\left(T_{3}\right)\Delta_{m}P_{k}\left(T_{2}\right)\right|,$$

that is, the triangle inequality holds.

For multiset-labeled rooted trees, the proof is similar and hence omitted.

**FIG. 9.**
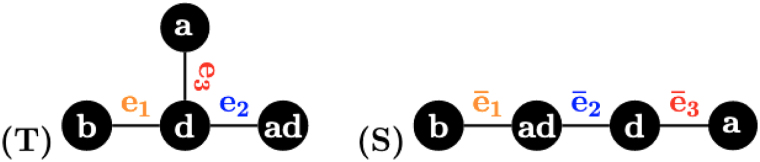
Two distinct multiset-labeled trees *S* and *T* satisfy that $P_{2}\left(S\right)=P_{2}\left(T\right)=\left\{\left\{a^{2}d^{2},b\right\},\left\{abd,ad\right\},\left\{a,abd^{2}\right\}\right\}$, showing that 2-RF score can be 0 even for distinct trees.

**Remark 3.** *For multiset-labeled trees,* $d_{k-RF}\left(S,T\right)=0$ *does not imply S and T are identical, as given in* Figure 9.

**Proposition 6.** *Let k* ≥ 0 *and S and T be two (rooted) trees whose nodes are labeled by* $L\left(S\right)$ *and* $L\left(T\right)$*, respectively. Then,* $d_{k-RF}\left(S,T\right)$ *can be computed in* 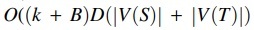 *time, where B is the maximum multiplicity of a label appearing in* $\left\{P_{T}\left(e,k\right)|e\in V\left(T\right)\right\}\cup \left\{P_{S}\left(e,k\right)|e\in V\left(S\right)\right\}$ *and* $D=\left|Supp\left(L\left(S\right)\right)\cup Supp\left(L\left(T\right)\right)\right|$.

*Proof.* An algorithm for the 1-labeled case can be modified as follows for computing *k*-RF on multiset-labeled rooted and unrooted trees:

- Represent each label multiset as a *D*-dimensional vector, in which the integer at position *j* is the multiplicity of the *j*-th label. Computing all edge-induced pairs in both trees takes $O\left(k\left(\left|E\left(S\right)\right|+\left|E\left(T\right)\right|\right)\right)$ set operations. Each set operation takes *D* integer operations.
- Radix-sort all the edge-induced pairs for *S* and *T* in $O\left(D\left(\left|E\left(S\right)\right|+B\right)\right)$ and $O\left(D\left(\left|E\left(T\right)\right|+B\right)\right)$ integer operations, respectively.
- Compute the symmetric difference of the set of the edge-induced pairs in the two input trees in $\left|E\left(S\right)\right|+\left|E\left(T\right)\right|$ set operation. Each set operation takes *D* integer operations.

In summary, one can compute $d_{k-RF}\left(S,T\right)$ using 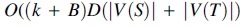 integer operations, as $\left|E\left(S\right)\right|=\left|V\left(S\right)\right|-1$.

### 5.4. Correlation of the *k*-RF and the other measures

Let *T* and *S* be two 1-labeled rooted trees with the same label set *X*. Again, we identify the nodes with their labels in the two trees. For any two subset *X*′ and *X*″ of *X*, we use $d_{J}\left(X\prime ,X\prime \prime \right)$ to denote their Jaccard distance. The CASet∩ distance between *T* and *S* is defined to be the average $d_{J}\left(A_{T}\left(i\right)\cap A_{T}\left(j\right),A_{S}\left(i\right)\cap A_{S}\left(j\right)\right)$ of a pair of nodes *i* and *j*, whereas the DISC∩ distance between *T* and *S* is the average $d_{J}\left(A_{T}\left(i\right)\setminus A_{T}\left(j\right),A_{S}\left(i\right)\setminus A_{S}\left(j\right)\right)$ of an order pair (*i,j*) of nodes DiNardo et al. (2020).

Using the Pearson correlation (PC), we compared the *k*-RF with CASet∩, DISC∩, and GRF (Llabrés et al., 2020) in the space of set-labeled trees for different *k* from 0 to 28.

**FIG. 10.**
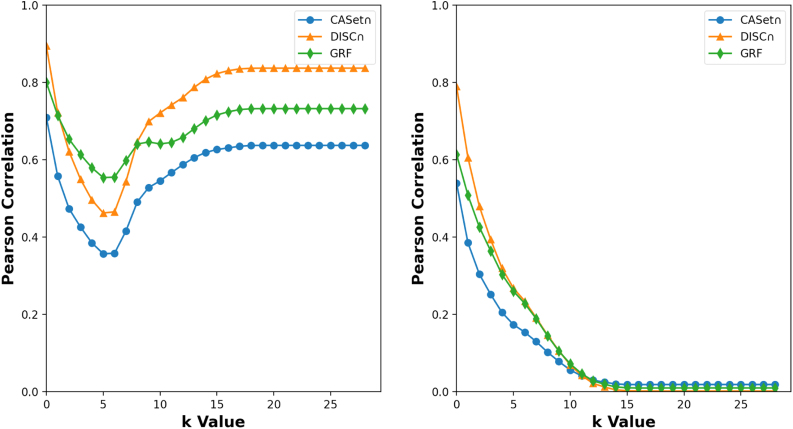
PC of the *k*-RF with CASet∩, DISC∩, and GRF. The analyses were conducted on rand rooted trees with the same label set (left) and with different but overlapping label sets (right) that were reported in Jahn et al. (2021). The PC became constant for *k ≥* 19 in the range *k*-RF becomes RF. CASet∩, Common Ancestor Set distance; DISC∩, Distinctly Inherited Set Comparison distance (DiNardo et al., 2020); GRF, Generalized RF distance (Llabrés et al., 2021).

First, we conducted the correlation analysis in the space of mutation trees with the same label set. Using a method reported by Jahn et al. (2021), we generated a simulated dataset containing 5000 rooted trees in which the root was labeled with 0 and the other nodes were labeled by the disjoint subsets of $\left\{1,2,\ldots ,29\right\}$, where the trees might have different number of nodes. Using all $\left(\begin{array}{c} 5,000\\ 2 \end{array}\right)$ pairwise scores for CASet∩, DISC∩, GRF, and *k*-RF, we conducted the PC analysis of *k*-RF with the other three (Fig. 10, left panel).

Our results show that CASet∩, DISC∩ and GRF were all positively correlated with *k*-RF. We observed the following facts:

- The GRF and *k*-RF are highly correlated for each *k < 8*.
- The DISC∩ and *k*-RF are highly correlated for each *k ≥ 8*.
- The 5-RF and 6-RF were less correlated to CASet∩, DISC∩ and GRF than other *k*-RF.
- The PC between *k*-RF and CASet∩ (respectively, DISC∩) increased when *k* went from 6 to 15.

Next, we performed PC analysis on trees characterized by distinct yet intersecting label sets. The dataset was created using the identical methodology, comprising a union of five sets of rooted trees, each encompassing 200 trees and sharing the same label set. Dissimilarity scores were calculated for each tree within the initial group and each tree in the remaining groups. Subsequently, we computed the PC between different dissimilarity values. Once more, all dissimilarity measures exhibited a positive correlation, although less pronounced than in the initial scenario [refer to Figure 10 (right)}. This observation could be because of that difference in label sets of two trees makes their *k*-RF score at least *k* + 1. However, the difference does not strongly contribute to the other distances because DISC∩ and CASet∩ consider the intersection of label sets (DiNardo et al., 2020), and GRF considers the intersection of clusters.

The right dotplot of Figure 10 shows that the *k*-RF and DISC∩ had the largest PC for *k* from 1 to 9, and the *k*-RF and the CASet∩ had the largest PC for *k* ≥ 10. Moreover, all the PCs decreased when *k* changed from 1 to 15. This trend was not observed in the first case. This decreasing trend could be the result of the fact that difference in label sets contributes to *k*-RF more as *k* increases.

## 6. CLUSTERING TREES WITH THE *k*-RF

A test was designed to compare the *k*-RF, CASet∩, DISC∩, and GRF in terms of clustering labeled trees.

We generated randomly 5 tree families each containing 50 trees using the program reported by Jahn et al. (2021). The nodes were labeled by the subsets of a set of size 30 in the trees of each family. The label sets of any two different families were distinct in only one label. We imposed such restriction on the label sets as in each tree, distinct nodes were labeled by disjoint subsets; hence, each different label between the label sets of two trees induces *d* pairs that only belong to the tree with the label, where *d* is the degree of the node with the label. Therefore, the more different the label sets are, the more distinguishable the trees could be by the *k*-RF.

We computed the pairwise dissimilarity scores for all 250 trees in the 5 families via each measure; we then clustered the 250 trees into *c* clusters using the *K*-means clustering method, where *c* ranges from 2 to 57. The clustering results were assessed using the Silhouette score (Kaufman and Rousseeuw, 2009).

**FIG. 11.**
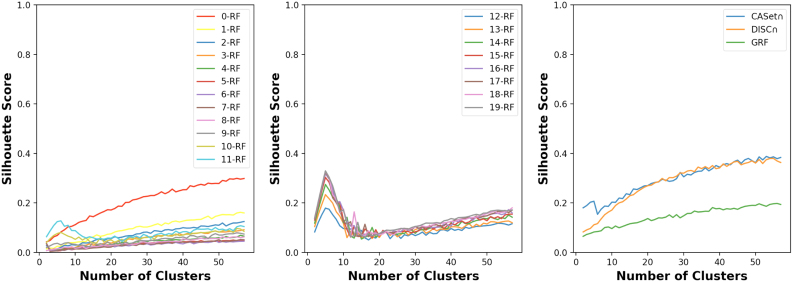
Silhouette scores of clustering 250 rooted trees with *k*-RF for 0≤k≤11(left) and 12≤k≤19(middle) and with CASet∩, DISC∩, and GRF (right).

As Figure 11 shows, the correct number of tree families was not recognized by either of the CASet∩, DISC∩, and GRF distances. However, among these three measures and the *k*-RF measures for *k ≤ 12*, the Silhouette score of CASet∩ was the highest value when the number of clusters was 5. Furthermore, the figure illustrates that the exact number of families was recognized by *k*-RF when *k* ranges from 12 to 19. Moreover, the Silhouette score of the *k* -RF increased when *k* increased from 8 to 19 This interesting observation may stem from the fact that as *k* increases, the number of pairs of trees achieving the highest possible *k*-RF score also increases, thereby enhancing the recognizability of families. It's worth noting that such pairs are guaranteed to exist when *k* is larger than the minimum diameter of the trees, which is 8 in our case.

## 7. CONCLUSIONS

The development of an efficient and robust measure for the comparison of labeled trees is important. In this study, we have proposed a novel variant of dissimilarity metrics, namely the *k*-RF, tailored for labeled trees. The *k*-RF facilitates the analysis of local structures in labeled trees, accommodating nodes labeled with (not necessarily the same) multisets. Significantly, these metrics find practical applicability in mutation trees used in cancer research.

The RF distance is succinctly expressed as (*n* − 1)-RF within the space of labeled trees with *n* nodes. By setting *k* to a value smaller than *n* − 1, the *k*-RF metric can capture analogous local regions in two labeled trees. Of note, for every *k*, the *k*-RF is a pseudometric for multiset-labeled trees and becomes a metric in the space of 1-labeled trees. However, the distribution of pairwise *k*-RF scores in the space of 1-labeled unrooted (or rooted) trees conforms to a Poisson distribution specifically for *k = n − 2*, and unlikely have the same trend for other values of *k* ≥ 1.

We verified the *k*-RF measures through a comprehensive comparison with CASet, DISC (DiNardo et al., 2020) and GRF (Llabrés et al., 2021) on randomly labeled trees generated by a house-made program (Jahn et al., 2021). Our findings revealed a consistent positive correlation between *k*-RF and each of the other three measures for every value of *k*. Of note, the correlation values exhibited a tendency to be higher when the measures were applied to assess mutation trees with identical label sets. Furthermore, our study underscored the superior clustering capabilities of *k*-RF compared with the three mentioned measures.

We would like to emphasize that selecting an appropriate *k*-RF in practical applications lacks a universal rule of thumb, primarily owing to a shortage of experience in this domain. Perhaps a judicious approach involves choosing a suitable *k*-RF by carefully considering the topological similarity among the trees under consideration.

Future work includes how to apply the *k*-RF to designing tree inference algorithms like GraPhyC (Govek et al., 2018) and also how to infer the exact frequency distribution of the *k*-RF for each *k* ≥ 1. It is also interesting to investigate the generalization of RF-distance for clonal trees (Llabrés et al., 2020).

The code for computing the pairwise *k*-RF scores of a group of multiset-labeled trees can be downloaded from https://github.com/Elahe-khayatian/k-RF-measures.git.
